# Prevalence of Second Victims, Risk Factors, and Support Strategies among German Nurses (SeViD-II Survey)

**DOI:** 10.3390/ijerph182010594

**Published:** 2021-10-10

**Authors:** Reinhard Strametz, Johannes C. Fendel, Peter Koch, Hannah Roesner, Max Zilezinski, Stefan Bushuven, Matthias Raspe

**Affiliations:** 1 Wiesbaden Business School, RheinMain University of Applied Sciences, 65183 Wiesbaden, Germany; hannah.roesner@hs-rm.de; 2 Medical Centre, Department for Psychosomatic Medicine and Psychotherapy, Medical Faculty, University of Freiburg, 79085 Freiburg, Germany; johannes.fendel@uniklinik-freiburg.de; 3 Centre of Excellence for Epidemiology and Health Services Research for Healthcare Professionals (CVcare), University Medical Centre Hamburg-Eppendorf, 20246 Hamburg, Germany; p.koch@uke.de; 4 WG Health Services Research|Hospital Care, Department of Internal Medicine, Faculty of Medicine, Martin-Luther-University Halle-Wittenberg, 06120 Halle (Saale), Germany; max.zilezinski@uk-halle.de; 5 Faculty of Medicine, Martin-Luther-University Halle-Wittenberg, Dorothea-Erxleben-Lernzentrum-Halle (DELH), Project FORMAT CONTINUUM, 06112 Halle (Saale), Germany; 6 Institute for Hospital Hygiene und Infection Prevention and Hegau-Jugendwerk Hospital Gailingen, Health Care Association District of Constance, 78315 Radolfzell, Germany; stefan.bushuven@glkn.de; 7 Institute for Medical Education, University Hospital, LMU Munich, 80336 Munich, Germany; 8 Department of Internal Medicine, Infectious Diseases and Respiratory Medicine, Charité—Universitätsmedizin Berlin, 10117 Berlin, Germany; matthias.raspe@charite.de; 9 Berlin Institute of Health, Humboldt-Universität zu Berlin, 10117 Berlin, Germany

**Keywords:** second victim, traumatisation, medical error, risk factors, support strategies

## Abstract

Background: Second victim phenomena (SVP) are critical to workplace and patient safety, and epidemiological data are limited to investigate the causes and impact on German health care. We investigated SVP in German nurses regarding prevalence, causes, and predisposition compared to a preceding study on German physicians (Second Victims in Deutschland/SeViD-I). Methods: We conducted a nationwide anonymous cross-sectional online study in 2020 using a modified SeViD questionnaire including the BFI-10 (personality traits). Statistical analysis was conducted using chi² tests and binary logistic regression models. Results: Of 332 nurses, 60% reported to experience SVP at least once a working lifetime, with a 12-month prevalence among SVP of 49%. Of the nurses, 24% reported recovery times of more than 1 year. In contrast to physicians from SeViD-I, a main cause for becoming a second victim was aggressive behavior by patients. High neuroticism values, higher age, and medium work life experience, but neither gender nor workplace position, were predisposing for SVP. Like SeViD-I, nurses reported demand for an institutional response in cases of SVP. Conclusions: SVP is common among German nurses and comprises other causes and a different course than in physicians. Further research should concentrate on specific prevention strategies, e.g., profession- and workplace-based educational programs.

## 1. Introduction

In this article, we report on the prevalence and recognition of the second victim phenomenon (SVP) and its risk factors in German nurses. To do so, we made use of the modified SeViD questionnaire (Second Victim in Deutschland) published recently [1].

The SVP was introduced by Wu “as the HCPs [health care professionals] who commit an error and are traumatized by the event manifesting psychological (shame, guilt, anxiety, grief, and depression), cognitive (compassion dissatisfaction, burnout, secondary traumatic stress), and/or physical reactions that have a personal negative impact” [2,3,4]. Scott and colleagues broadened the SVP definition in 2009, and defined second victims as “healthcare providers who are involved in an unanticipated adverse patient event, in a medical error and/or a patient related injury and become victimized in the sense that the provider is traumatized by the event” [5]. Though ethically debated for its terminology [6], there is rising scientific [7] and political [8] interest in this prevalent phenomenon [9]. SVP puts patients [10], health care providers, and medico-economical systems at risk for losing valuable individuals or their workforce due to stress, defensive medicine [11], depression, post-traumatic stress disorder (PTSD) [4,7] and even suicide [12]. Growing efforts comprise the development of screening questionnaires and support tools in different languages [13,14,15,16], as well as structured narrative and systematics reviews about the effects and coping strategies [17,18,19].

Detection and awareness for the SVP at all operational levels are the basis for building safer environments and a long-lasting establishment of self-care. This is best known in palliative medicine [20] and care-ethics [21]. Furthermore, caring for oneself and for others concerning adverse effects of critical incidents and medical error is an essential part of modern professional and leadership competencies [22,23].

In 2019, our working group investigated the impact of SVP on young German post-graduate physicians in internal medicine [1], using the previously designed and validated 46-item SeViD (Second Victim in Deutschland/Second Victim in Germany) questionnaire [24]. Aside from demographic data, the SEVID-I questionnaire consists of three domains (“general experience”, “SVP-Symptoms”, and “SVP support strategies”). In this project, we addressed 555 internal medicine physicians under 35 years of age. We were able to show that 59% experienced one or more instance of SVP in their career, and 35% within the last 12 months. Further, 12% of the participants reported not to have recovered from an SVP within a year or not to have recovered at all. Females were affected more often and with higher burden. Additionally, we detected inter-individual differences that were possibly linked to psychological traits.

However, the impact of SVP on other professional groups and disciplines in Germany, such as nurses, has not been investigated so far. To close this gap, we adapted the hypotheses and methodology of SeViD-I in a successor project named SeViD-II.

In SeViD-II, we investigated the recent recognition of SVP and its prevalence, as well as supporting strategies in nurses and associated assistants. Moreover, we assessed intercorrelation and subgroups effects, and investigated associations with certain personality traits [25] (i.e., openness, conscientiousness, extraversion, agreeableness, and neuroticism) [26].

We hypothesized:

1. SVP is associated with comparably high prevalence in a convenience sample of German nurses as in young German physicians in internal medicine (SeViD-I study).
2. SVP is associated with certain risk factors in detail:
  2.1. Age.
  2.2. Working experience.
  2.3. Female gender.
3. SVP is associated with certain personality traits, in detail:
  3.1. Openness is negatively related to probability and symptom load of SVP.
  3.2. Conscientiousness is negatively related to probability and symptom load of SVP.
  3.3. Extraversion is negatively related to probability and symptom load of SVP.
  3.4. Agreeableness is negatively related to probability and symptom load of SVP.
  3.5. Neuroticism is positively related to probability and symptom load of SVP.
4. Second victims favor similar support strategies as the physicians did in SeViD-I.

## 2. Materials and Methods

### 2.1. Construction and Validation of the SeViD Questionnaire

The detailed construction and validation of the questionnaire is described elsewhere [24]. Since this original publication is in the German language, a brief description is given here: we identified existing questionnaires evaluating the second victim phenomenon in healthcare, which were identified by a systematic literature search. Based on these sources (six questionnaires related to nine resources), we developed a new German-language questionnaire. This questionnaire was tailored to our prespecified needs in terms of brevity and easy applicability on different groups of healthcare professionals in European healthcare systems, and covered broad aspects of the second victim’s phenomenon (prevalence, symptoms, and support strategies). The preliminary version of this questionnaire was subject to cognitive pretesting to ensure content validity. We included healthcare professionals of different professional groups with or without previous second victim experience to participate as volunteers for all pre-tests after informed consent. All cognitive pre-tests were conducted by an independent researcher. The final questionnaire consisted of three domains and 40 items (Table 1). For the symptoms, domain participants answered by a 3-point ordinal scale (strongly pronounced, weakly pronounced, not pronounced), and for the support strategies domain, by a 4-point ordinal scale (very helpful, rather helpful, rather not helpful, and not helpful). The options “Don’t know” and “I cannot judge this”, respectively, were also included.

### 2.2. Design and Conduction of the SeViD-II Survey

The report of the SeViD-II survey adheres to the checklist for reporting results of internet e-surveys (CHERRIES) [27]. The survey was conducted using the commercial application Momentive^®^ (San Mateo, CA, USA). An invitation with a link for participation was sent via the newsletters and social media platforms of the German Nurses Association (Deutscher Berufsverband für Pflegeberufe/DBfK e.V.). The study period was from the 5th of October to the 13th of December, 2020 (10 weeks). Reminders were sent after four and eight weeks. Beforehand, the local regulation authority confirmed that that a formal official ethical approval was not necessary. Data collection was completely anonymized with neither tokens, cookies, nor IP addresses stored. The presentation of the survey was split into six different screen pages. The invitation and reminder for participation included statements on the purpose of the survey, an explanation of the term second victim, information on responsible investigators (including a contact email address in case of questions), information on the anonymity of the study, length, voluntary participation, and data protection. The survey was only accessible with the provided link. Because of the complete anonymization, potential participation more than once and spread of the invitation link to others outside the target population could not be controlled. Ten items assessing baseline characteristics were added to the above described SeViD questionnaire, including gender, age, formal education, work experience, leading position, working full- or part time, working place, working mode (in terms of hours/shifts), time during the last 12 months in patient care, and number of beds in hospital. One additional question assessed whether the key traumatizing incident was connected to the SARS-CoV-2 pandemic. Finally, the validated Big Five Inventory (BFI)-10 was applied, which allows a rough measurement of the individual personality structure on five dimensions: openness; conscientiousness; extraversion; agreeableness; and neuroticism (two items each; 5-point ordinal scale between “strongly agree” and “strongly disagree”) [26]. The survey used adaptive questioning (i.e., questions of the symptoms domain appeared only to participants who had experienced second victim incidents), and one question had to be answered before moving to the next. Participants were able to move backwards to change their answers to previous questions. Before closing the survey, participants could leave comments. All available data were analyzed for each question, with specific numbers of responding participants indicated.

### 2.3. Preparation and Re-Coding of Variables for Statistical Analysis

The continuous variables age and working experience were categorized in three equal groups by number of participants (≤31, 32–43, and 44–66 years of age, and ≤7, 8–20, and 21–46 years of working experience, respectively). The item asking for the place of work in the hospital was dichotomized in working predominantly in acute care (intensive/intermediate care and/or emergency department) vs. others. Other dichotomizations were performed for the second victim status (having experienced one/several second victim incidents vs. never experienced such incidents) and time to self-perceived full recovery after the key incident (one month or less vs. more than one month). For an estimation of the symptom load, a sum score was calculated. Answers “strongly pronounced” of the symptom domain were counted as 1, and “weakly pronounced” as 0.5 (“not at all” and “don’t know” as 0). Following this, a sum score for each participant was calculated. Based on the median, this new variable was dichotomized for establishing a low (≤7) and high (≥7.5) symptom load group, or it was categorized in three equal groups by number of participants (scores of ≤6, 6.5–8.5, and 9.0–17.5). For each dimension of the BFI-10, mean scores were calculated (“strongly agree” = 5 and “strongly disagree” = 1; for each dimension, one item with positive and one item with negative polarity; items with negative polarity were inverted before calculating mean scores). Based on the median, a group of higher and lower scores was formed for each dimension.

### 2.4. Statistics

Expected and observed distribution patterns were compared using contingency tables, and analyzed for statistical significance by applying the chi² test. The influence of the independent variables (gender, age, working experience, leading position, workplace, symptoms score, and personality dimensions; with different combinations of variables for each model) on the dependent variables (being a second victim, symptom load, and time to self-perceived recovery) were assessed using binary logistic regression models. All statistical analyses were performed with SPSS Statistics Version 26 (IBM, New York, NY, USA).

## 3. Results

### 3.1. Baseline Characteristics

Over the study period, 332 participants took part in the survey. Of the participants, 86% (286/332) completed the whole questionnaire. All baseline characteristics are shown in Table 2.

### 3.2. Second Victim Incidents

**Figure 1:**
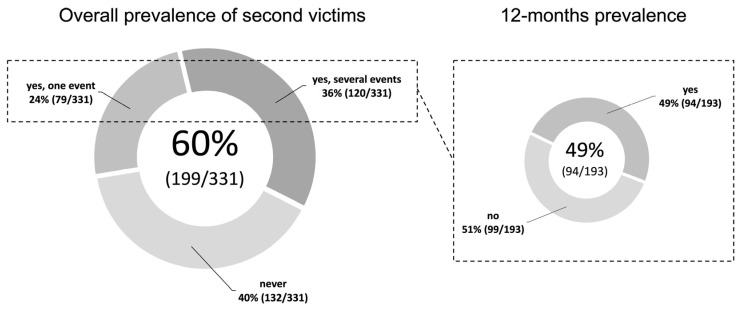
Overall and 12-months prevalence of second victims.

Of the participants surveyed, 75% (249/331) had no knowledge of the term “second victim” before participation in this survey. The prevalence of second victims is shown in Figure 1. The 12-months prevalence among all study participants was 28% (94/332).

The types of key incidents and self-perceived time until full recovery is displayed in Table 3. Unexpected death/suicide of a patient (29% (56/193)), aggressively acting patient or relatives (25% (49/193)), and events with patient harm (24% (47/193)) were reported as most frequent key events. Self-perceived time to full recovery after the key event was reported as up to one month by 59% (105/177), and as more than one month or never by 41% (72/177) of the second victims. Support came in 49% (88/181) from colleagues, in 23% (41/181) from friends and relatives, in 17% (30/181) from supervisors, in 9% (16/181) from professionals (psychiatrists, psychologists), and in 3% (6/181) from administration.

Moreover, 9% (16/177) of the traumatized participants reported that the key incident was connected to the SARS-CoV-2 pandemic. Sum scores for the five BFI-10 dimensions were (mean± standard deviation; range 1–5; *n* = 286 each): openness 3.5 ± 1; conscientiousness 4.2 ± 0.8; extraversion 3.5 ± 0.9; agreeableness 3.3 ± 0.8; and neuroticism 2.7 ± 0.9.

### 3.3. Risks Factors for Being a Second Victim

Based on a binary logistic regression model, the influence of the independent variables gender, age, working experience, leading position, workplace in acute care, and the five personality dimensions on being a second victim was analyzed (*n* = 286; Table 4). A statistically significant risk factor found in this model was pronounced neuroticism, with an odds ratio of 2.77 (95%-CI 1.59–4.84, *p* < 0.01).

### 3.4. Factors with Impact on the Symptom Load of Second Victims

Based on a binary logistic regression model, the influence of the independent variables gender, age, working experience, leading position, workplace in acute care, and the five personality dimensions on the symptom load of second victims was analyzed (*n* = 170; Table 5). No statistically significant risk factors were found.

### 3.5. Factors with Impact on the Time to Self-Perceived Full Recovery after the Key Incident

In a third binary logistic regression model, the influence of the independent variables gender, age, working experience, leading position, workplace in acute care, symptom score, and the five personality dimensions on the self-perceived time to full recovery (one month or less vs. more than one month) of second victims was analyzed (*n* = 170; Table A1). Statistically significant risk factors for a time to full recovery of more than one month after the key traumatizing incident were: being among the highest age group (44–66 years of age), with an odds ratio of 9.16 (95%-CI 1.91–43.9, *p* = 0.01); being among the middle working experience group (8–20 years), with an odds ratio of 3.66 (95%-CI 1.06–12.7, *p* = 0.04); and participants with a pronounced openness personality dimension, with an odds ratio of 2.5 (95%-CI 1.13–5.50, *p* = 0.02).

### 3.6. Support Strategies for Second Victims

The participants (*n* = 290) were asked to rate 13 established support strategies for second victims (Table 6). Support measures rated by > 90% of the second victims as “very” or “rather helpful” were: the possibility to discuss emotional and ethical issues (91% (157/173) and prompt debriefing/crisis intervention after the incident (91% (157/173). The ratings of support strategies by second victims vs. others were tested for unequal distributions (chi² tests; 4-point Likert scale). Statistically significant differences were only observed for the support strategy “immediate time out to recover”, which was rated more often as “very” or “rather helpful” among the group of no second victims (82% (96/117) vs. 67% (116/173), *p* = 0.01).

## 4. Discussion

To the best of our knowledge, this is the first study investigating the prevalence and characteristics of the SVP among German nurses, and associating it with personality traits. In this investigation, we could confirm or reject our hypotheses as follows.

First: The SVP is comparably prevalent in a convenience sample of German nurses as in young German physicians in internal medicine.

Our findings show a prevalence of 59% among nurses experiencing SVP at least once a lifetime. This is comparable to young physicians who showed 60% in the preceding study [1]. The prevalence in our sample is higher than in other countries reporting 10 % to 48% [9], raising further questions on the epidemiology of the phenomenon. However, these lower numbers in our studies were pre-SARS-CoV-2-pandemic, and the role of the pandemic could not be clarified extensively. In our study, 9% of all participants reported that SVP was related to the pandemic, but it is unclear if this percentage occurred additionally or not. Further, we do not know exactly how the pandemic influenced SVP occurrence. However, first evidence indicates that SVP plays a significant role in 2020 and 2021 [28,29,30,31]. Combined with our findings that 24% of the affected nurses report recovery times of more than 12 months, these data are alarming. SVP events might have a substantial and long-lasting impact through causing risks for patients, other health care providers, and eventually, whole health care systems, and the economy in general.

Physicians in SeViD-I reported a long-lasting effect in 12% of all cases. Although these lower reporting rates may be affected by social expectations [32] or suffering in silence due to the physicians’ self-image [33], higher rates in nurses may be explained by more frequent exposure to stressful situations compared to physicians. Remarkably, nurses experienced SVP more often due to aggressive patients (25%) than physicians did (15%). It is well known that nurses have more intense contact to patients. As a surrogate, nurses experience more than three-fold indications for hand hygiene in daily practice compared to physicians [34], have a lower professional-to-patient ratio, and more frequently face verbal and physical aggressive behavior in daily care [35], perhaps due to perceived or actual social vulnerability and lack of respect [36]. In contrast, suicide of patients and harmful adverse events showed to be of higher emphasis in young physicians (35% and 34% respectively) than in nurses (29% and 24% respectively).

In summary, our data suggest that the prevalence of SVP is comparable among German nurses and physicians. However, the origin leading to the phenomenon is different for these groups. The differences in causes and effects of SVP among the two professions are of value for crew resource managers, educators, and team leaders to focus on tailored coping strategies of adverse events [37].

Demographic differences showed that the physicians in SeViD-I were younger than the nurses (32 vs. 38 years), and women were more prevalent in SeViD-II (74% vs. 65%). Gender distribution showed to be valid according to German data on gender demographics among health care providers [38,39]. In contrast to physicians, nurses seem to experience SVP longer than physicians, indicating a higher and longer lasting impact on patient safety and professional well-being. Consequently, high medical and economic burden for the whole health care system can be expected.

Second and third: Similar risk factors can be identified compared to SeViD-I, and the SVP correlates to personality traits measured by the BFI-10 inventory.

In SeViD-I, our working group showed that the female sex was associated with a 2.5-fold risk to become a second victim, and with a two-fold risk to develop a high symptom load. Additionally, time to full recovery was more likely if health care professionals worked in an acute care setting (OR 0.5).

In addition to these findings, other risk factors could be identified in SeViD-II. A high neuroticism score in the BFI-10 showed to have an OR of 2.77 for becoming a second victim. Moreover, a higher age (OR 9.16), middle working experience (OR 3.66), and a pronounced openness (OR 2.5) were predictors for a longer time to self-perceived full recovery. However, in SeViD-II, no factors were predicting the magnitude of symptoms, and gender was not identified as a risk factor.

BFI-10, working experience, and higher age were not assessed in SeViD-I comparably, so there was no possibility to affirm our hypothesis on these three issues. Nevertheless, the finding of association between neuroticism and risk for SVP must be acknowledged. High neuroticism values are associated with perfectionism [40] and a rather poor ability to cope with stressful situations [41,42]. Maladaptive perfectionism is very common both in young physicians [43] and nurses [44], and therefore, could explain the risks for SVP. Moreover, perfectionism has been linked to mental disorders like depression [45,46]. Likewise, neuroticism and resilience were correlated negatively in other settings [47], and high neuroticism is associated with high symptom load and length of PTSD [48,49]. These findings call for further studies on both the association of personality traits to the risk of SVP, as well as the differentiation between SVP and other psychological reactions after stressful experiences (e.g., burnout, PTSD, depression).

Pronounced openness, which predicts longer time to self-perceived full recovery, seems counterintuitive at first. An explanatory hypothesis could be, that HCPs with pronounced openness are more likely to expose themselves to difficult and, therefore, potentially traumatizing treatment situations. Furthermore, these participants could have more likely taken part in this survey and might have answered more truthfully.

However, personality traits seem to play a major role in SVP, indicating again the relevance of different and adaptive prevention, screening, and coping strategies for health care providers. Our findings indicate the need for research on whether special situations leading to SVP may be linked to different scores in personality testing or not. Aside from personal traits, other aspects of diversity (age, gender, religion, ethnicity, religion), as well as qualification and work/life experience should be addressed in further projects.

In summary, personal factors of health care providers, such as female sex and high neuroticism, put many health care providers at risk for SVP and other psychological phenomena. This affirms the urgency to address SVP early in recruitment and medical education, as well as during and after service in health care systems.

Fourth: Second victims favor similar support strategies as the physicians did in SeViD-I.

Support strategies rated by 90% or more of the participants to be “rather” or “very helpful” when experiencing the second victim phenomenon were particularly taken into consideration. Comparing support strategies in SeViD-I and -II, the opportunity to discuss emotional and ethical issues (93 % vs. 91%), and prompt debriefing/crisis intervention after an incident (95% vs. 91%) were among the top three measures among both physicians and nurses. Thus, this hypothesis could be confirmed. The results indicate that, in contrast to events leading to SVP, intervention strategies after experiencing SVP may not depend on the profession, according to our data. For supervisors and leaders, this could lead to uniform, multiprofessional, or interprofessional effective intervention programs for “rapid response to SVP”. Furthermore, interventional strategies are most relevant to undergraduate and postgraduate medical education, competency building [50], and life-long learning [51] in order to establish long lasting cultures of safety. Educators are expected to transfer factual knowledge about the SVP (“What is a second victim effect?”), communicative psycho-motoric skills (“How do I speak to a colleague experiencing the SVP?”), problem solving strategies (“What can I do if a colleague gets into PTSD or even gets suicidal?”), an attitude (“the mindset”) towards SVP (“Commitment of SVP symptoms is a strength, not a personal weakness!”), and the actual behavior of health care providers and supervisors in volatile, uncertain, complex, and ambiguous work place environments [52].

However, the selective sample limits the generalizability of the results, and indicate further research for clarification of this aspect in other medical settings and professions.

Our findings may be limited in several ways. First, the cross-sectional design can describe associations, but will never link causation. Second, the explored data is based on a convenience sample that is liable to selection bias. However, it has several positive aspects concerning completer rates [53]. Hence, representativity may be debated, and further studies in closed environments and with larger sample sizes are warranted [54]. Recently, personality traits among 518 nurses from the Heidelberg University Hospital in Germany were studied in a cross-sectional survey, by means of the BFI-10. The values were very similar compared to our study, indicating good representativity of our sample with regard to this aspect (SeViD-II vs. Heidelberg sample: openness 3.5 ± 1 vs. 3.5 ± 1, conscientiousness 4.2 ± 0.8 vs. 4.1 ± 0.8, extraversion 3.5 ± 0.9 vs. 3.5 ± 1, agreeableness 3.3 ± 0.8 vs. 3.4 ± 0.8, and neuroticism 2.7 ± 0.9 vs. 2.7 ± 0.9) [55]. Third, the DBfK database did not allow for analysis of the target population, thus, a non-responder analysis could not be conducted. More nurses with traumatic incidents in their past could have taken advantage of the survey, however, responding to surveys and completing them depend on several other factors [56]. Fourth, investigating only members of one professional association could harbor bias because members could have specific characteristics that might distinguish them from others. Fifth, another limitation is the number of dropouts. The fear of potential participants to admit that something went wrong could have negatively influenced the response rate. There is often still a culture of blame in the workplace and fear of recrimination [33]. Sixth, due to the anonymous conduction of the survey, we cannot exclude the possibility of multiple participations of certain participants. Seventh, the item “time to full recovery” is difficult to define and prone to recall bias. Participants might feel that they have fully recovered, but the traumatic incident could still influence their behavior. Additionally, recovery might be a process with ups and downs [3]. Last, our analysis is mainly explorative. Likewise, it is supposed to generate further hypotheses and a basis for more research on this important topic. Authors should discuss the results and how they can be interpreted from the perspective of previous studies and of the working hypotheses. The findings and their implications should be discussed in the broadest context possible. Future research directions may also be highlighted.

## 5. Conclusions

In this study, we could confirm that SVP is highly prevalent among German nurses. Causes of SVP differed significantly from physicians, and the time to self-perceived recovery was substantially longer. The personality traits neuroticism and openness were associated with a higher risk for SVP (in terms of becoming a second victim and the time to recovery, respectively). Middle work experience and higher age were other new risk factors described in this cohort. Other risk factors that were found in SeViD-I could not be detected in SeViD-II. However, first responses when facing SVP were similar for nurses and physicians. Despite the possibility of certain biases (e.g., sample size and the advantages and disadvantages of convenience sampling), this study contributes to a better understanding of the SVP in nurses and young physicians. Further research is needed to differentiate SVP from other psychological phenomena after critical incidents, and to clarify the role of personality traits, age, work experience, and other aspects of diversity (gender, age, disability, religion, ethnicity), and other circumstances (such as the pandemic) in prevention, rapid response management, coping strategies, and persistence of the SVP. Additionally, more research would be desirable, focusing on other under- and postgraduate health care providers (e.g., paramedics, psychologists, medical assistants, midwifes), as well as their sub-specializations.

## Figures and Tables

**Table 1:** Domains and items of the SeViD questionnaire.

Domain	Item
General experience with second victim phenomenon	Knowledge of the term second victim
	Lifetime prevalence of second victim experience
	12-month prevalence of second victim experience
	Type of key incident
	Seek for support after key incident
	Types of groups supporting after key incident
	Self-perceived time to full recovery after key incident
Second victim symptoms ^1^	Fear of social exclusion from colleagues
	Fear of losing the job
	Lethargy
	Depressed mood
	Concentration problems
	Reactivation of situation outside job site
	Reactivation of situation at job site
	Aggressive, risky behavior
	Defensive, overprotective behavior
	Psychosomatic reactions (headaches, back pain)
	Difficulties to sleep or excessive need to sleep
	Use of substances (alcohol/drugs) due to this event
	Sense of shame
	Feelings of guilt
	Lower self-confidence
	Social isolation
	Anger against others
	Anger against oneself
	Desire to get support from others
	Desire to work through the incident for deeper understanding
Second victim support strategies	Immediate time out to recover
	Access to counselling, including psychological/psychiatric services
	Possibility to discuss emotional and ethical issues
	Clear information about processes (e.g., root cause analysis, incident reporting)
	Formal peer to peer support
	Informal emotional support
	Prompt debriefing/crisis intervention
	Supportive guidance for continuing clinical duties
	Help to communicate with patients
	Clear guidance about the roles to be expected after the incident
	Help to actively participate to work through this incident
	Safe opportunity to contribute insights to prevent similar events in future
	Opportunity to seek for legal advice after an incident

^1^ listed in alphabetical order of the German version of the questionnaire.

**Table 2:** Baseline characteristics of the study participants.

Baseline Characteristics		
Total number of participants		332
Gender (female: male: diverse)		73.8% (245): 24.7% (82): 1.5% (5)
Age (years)	Mean ± SD	38.74 ± 11.46
	≤31	32.5% (108)
	32–43	31.9% (106)
	44–66	35.5% (118)
Formal education (years of training)	Nurses (3)	92.2% (306)
	Nursing assistants (1)	4.8% (16)
	Others	3% (10)
Work experience (years)	Mean ± SD	15.6 ± 11.41
Leading position		35.5% (118)
Full: part time		63.3% (210): 36.7% (122)
Place of work ^1^	Ward	37.7% (125)
	IMC/ICU	32.2% (107)
	Interven./diagn.	5.7% (19)
	Emergency Dpt.	5.4% (18)
	Rehabilitation	3.0% (10)
	Day clinic	0.6% (2)
	Short-term care	0.6% (2)
	Other	24.1% (80)
Working mode (time)	Shifts with nights	53.3% (177)
	Same time each day	15.7% (52)
	Shifts, no nights	13.3% (44)
	Irregular, no shifts	11.4% (38)
	Only night shifts	2.1% (7)
	Other mode	4.2% (14)
Months during last year in PC	Mean ± SD	9 ± 4
Number of beds in hospital	≤99	5.7% (19)
	100–299	17.2% (57)
	300–599	32.8% (109)
	≥600	38.3% (127)
	Not known	6.0% (20)
Openness	Mean ± SD	3.47 ± 0.95
Conscientiousness	Mean ± SD	4.23 ± 0.79
Extraversion	Mean ± SD	3.48 ± 0.87
Agreeableness	Mean ± SD	3.25 ± 0.78
Neuroticism	Mean ± SD	2.67 ± 0.85

Each item was answered by all 332 participants of the study. Ass., assistant; SD, standard deviation; IMC, intermediate care; ICU, intensive care unit; Interven./diagn., unit for interventional and diagnostic procedures; Dpt., department; PC, patient care; ^1^ participants could choose more than one option.

**Table 3:** Type of key events and time to self-perceived full recovery among second victims.

**Type of Key Incident (*n* = 193)**	**%**	***n***
Unexpected death/suicide of a patient	29	56
Aggressively acting patient or relatives	25	49
Event with patient harm	24	47
Near miss	12	24
Unexpected death/suicide of a colleague	6	11
Other types	3	6
**Self-perceived time to full recovery after key incident (*n* = 177)**	**%**	***n***
Less than one day	5	8
Within one week	23	41
Within one month	32	56
Within one year	18	31
More than one year	10	17
Never	14	24

**Table 4:** Risk factors for being a second victim.

*n* = 286		Having Experienced One/Several Second Victim Incidents *n* = 170 (59%)			
Independent Variable		Final Model r^2^ = 0.131 ^1^			
		ReCoB ^2^	*p*	Odds Ratio ^3^	95%-CI ^4^
**Gender ^5^**	female (*n* = 214)				
	male (*n* = 69)	−0.28	0.39	0.76	0.41–1.41
	diverse (*n* = 3)	0.09	0.94	1.09	0.09–12.78
**Age ^6^** (years)	≤31 (*n* = 93)				
	32–43 (*n* = 91)	0.19	0.62	1.21	0.57–2.57
	44–66 (*n* = 102)	0.81	0.17	2.25	0.71–7.09
**Work experience ^7^** (years)	0–7 (*n* = 95)				
	8–20 (*n* = 101)	0.18	0.64	1.19	0.57–2.48
	21–46 (*n* = 90)	0.20	0.75	1.22	0.37–4.08
**Leading position ^8^** (*n* = 103)		−0.22	0.47	0.81	0.45–1.45
**Workplace in acute care ^9^** (*n* = 105)		−0.28	0.31	0.76	0.45–1.29
**Openness ^10^**	≤3 (*n* = 117)				
	3.5–5 (*n* = 169)	0.17	0.52	1.19	0.71–1.99
**Conscientiousness ^10^**	≤4 (*n* = 121)				
	4.5–5 (*n* = 165)	−0.21	0.45	0.81	0.47–1.39
**Extraversion ^10^**	≤3 (*n* = 120)				
	3.5–5 (*n* = 166)	0.52	0.06	1.69	0.99–2.88
**Agreeableness ^10^**	≤3 (*n* = 138)				
	3.5–5 (*n* = 148)	−0.11	0.68	0.90	0.54–1.50
**Neuroticism ^10^**	≤2.5 (*n* = 162)				
	3–5 (*n* = 124)	1.02	<0.01	2.77	1.59–4.84

For the binary logistic regression model, the dependent variable second victim status was set to never been a second victim vs. having experienced one or several second victim incidents. ^1^ Nagelkerkes r2; ^2^ regression coefficient B; ^3^ exponentiation of the B coefficient (Exp(B)) or odds ratio; ^4^ confidence interval; ^5^ reference category (RC) is female sex; ^6^ RC is ≤31 years of age; ^7^ RC is 0–7 years of working experience; ^8^ RC is no leading position; ^9^ RC is not working in acute care (predominantly in ICU and/or emergency department); ^10^ RC are lower sum scores based on median splits.

**Table 5:** Factors influencing the symptom load of second victims.

*n* = 170		High Symptom Load of Second Victims *n* = 95 (56%)			
Independent Variable		Final Model r^2^ = 0.131 ^1^			
		ReCoB ^2^	*p*	Odds Ratio ^3^	95%-CI ^4^
Gender ^5^	female (*n* = 133)				
	male (*n* = 35)	−0.19	0.67	0.83	0.35–1.95
	diverse (*n* = 2)	−0.77	0.61	0.46	0.03–8.52
Age ^6^ (years)	≤31 (*n* = 47)				
	32–43 (*n* = 52)	0.76	0.19	2.13	0.69–6.58
	44–66 (*n* = 71)	0.28	0.70	1.32	0.33–5.32
Work experience ^7^ (years)	0–7 (*n* = 50)				
	8–20 (*n* = 58)	−1.00	0.07	0.37	0.12–1.10
	21–46 (*n* = 62)	−0.49	0.51	0.61	0.14–2.63
Leading position ^8^ (*n* = 63)		−0.34	0.36	0.71	0.35–1.46
Workplace in acute care ^9^ (*n* = 57)		−0.23	0.54	0.80	0.39–1.64
Openness ^10^	≤3 (*n* = 67)				
	3.5–5 (*n* = 103)	0.31	0.38	1.36	0.69–2.70
Conscientiousness ^10^	≤4 (*n* = 76)				
	4.5–5 (*n* = 94)	−0.23	0.51	0.80	0.41–1.57
Extraversion ^10^	≤3 (*n* = 67)				
	3.5–5 (*n* = 103)	−0.50	0.16	0.61	0.30–1.23
Agreeableness ^10^	≤3 (*n* = 83)				
	3.5–5 (*n* = 87)	−0.54	0.12	0.58	0.30–1.14
Neuroticism ^10^	≤2.5 (*n* = 81)				
	3–5 (*n* = 89)	0.49	0.17	1.63	0.82–3.26

For the construction of the symptom load score, see methods section. For this binary logistic regression model, the symptom score was split based on its median in two groups with lower (≤ 7 points) vs. higher (≥ 7.5 points) symptom load scores. ^1^ Nagelkerkes r^2^; ^2^ regression coefficient B; ^3^ exponentiation of the B coefficient (Exp(B)) or odds ratio; ^4^ confidence interval; ^5^ reference category (RC) is female sex; ^6^ RC is ≤31 years of age; ^7^ RC is 0–7 years of working experience; ^8^ RC is no leading position; ^9^ RC is not working in acute care (predominantly in ICU and/or emergency department); ^10^ RC are lower sum scores based on median splits.

**Table 6:** Rating of support strategies by participants with and without having experienced second victim incidents.

Support Strategy	No Second Victims (*n* = 117)		Second Victims (*n* = 173)		*p* (chi²)
	Rated Rather or very Helpful	Rated Rather not or not Helpful	Rated Rather or very Helpful	Rated Rather not or not Helpful	
	% (*n*)	% (*n*)	% (*n*)	% (*n*)	
1. Immediate time out to recover	82 (96)	12 (14)	67 (116)	25 (44)	0.01
2. Access to counselling including psychological/psychiatric services	92 (108)	4 (5)	87 (150)	7 (12)	0.32
3. Opportunity to discuss emotional and ethical issues	91 (106)	6 (7)	91 (157)	6 (11)	0.96
4. Clear information about processes (e.g., root cause analysis, incident reporting)	86 (100)	11 (13)	84 (145)	13 (23)	0.84
5. Formal peer to peer support	80 (94)	10 (12)	84 (145)	12 (20)	0.27
6. Informal emotional support	73 (85)	18 (21)	72 (125)	20 (34)	0.88
7. Prompt debriefing/crisis intervention	92 (108)	3 (4)	91 (157)	5 (9)	0.77
8. Supportive guidance for continuing clinical duties	72 (84)	18 (21)	69 (120)	23 (39)	0.57
9. Help to communicate with patients	79 (92)	14 (16)	70 (121)	24 (41)	0.42
10. Clear guidance about the roles to be expected after the incident	74 (87)	21 (25)	70 (121)	22 (38)	0.42
11. Help to actively participate to work through this incident	93 (109)	3 (3)	86 (149)	8 (14)	0.12
12. Safe opportunity to contribute insights to prevent similar events in future	91 (106)	6 (7)	86 (148)	8 (14)	0.41

## Data Availability

The data presented in this study are available on request from the corresponding author.

## Appendix A

**Table A1:** Factors influencing the time to self-perceived full recovery.

*n* = 170		Time to Full Recovery > 1 Month *n* = 70 (41%)			
Independent Variable		Final Model r^2^ = 0.371 ^1^			
		ReCoB ^2^	*p*	Odds Ratio ^3^	95%-CI ^4^
Gender ^5^	female (*n* = 133)				
	male (*n* = 35)	0.21	0.66	1.24	0.48–3.17
	diverse (*n* = 2)	23.5	0.99	--- *	--- *
Age ^6^ (years)	≤31 (*n* = 47)				
	32–43 (*n* = 52)	1.13	0.09	3.11	0.83–11.6
	44–66 (*n* = 71)	2.21	0.01	9.16	1.91–43.9
Work experience ^7^ (years)	0–7 (*n* = 50)				
	8–20 (*n* = 58)	1.30	0.04	3.66	1.06–12.7
	21–46 (*n* = 62)	0.61	0.43	1.85	0.40–8.51
Leading position ^8^ (*n* = 63)		0.20	0.63	1.22	0.54–2.76
Workplace in acute care ^9^ (*n* = 57)		−0.02	0.96	0.98	0.43–2.20
Symptom score ^10^	≤6 (*n* = 52)				
	6.5–8.5 (*n* = 54)	−0.07	0.89	0.94	0.36–2.46
	9–17.5 (*n* = 64)	0.88	0.06	2.41	0.97–6.02
Openness ^11^	≤3 (*n* = 67)				
	3.5–5 (*n* = 103)	0.91	0.02	2.50	1.13–5.50
Conscientiousness ^11^	≤4 (*n* = 76)				
	4.5–5 (*n* = 94)	0.08	0.85	1.08	0.50–2.33
Extraversion ^11^	≤3 (*n* = 67)				
	3.5–5 (*n* = 103)	−0.41	0.30	0.67	0.31–1.45
Agreeableness ^11^	≤3 (*n* = 83)				
	3.5–5 (*n* = 87)	−0.42	0.29	0.66	0.30–1.43
Neuroticism ^11^	≤2.5 (*n* = 81)				
	3–5 (*n* = 89)	0.38	0.36	1.47	0.64–3.35

For the binary logistic regression model, the dependent variable time to full recovery was set to up to one month vs. more than one month. ^1^ Nagelkerkes r^2^; ^2^ regression coefficient B; ^3^ exponentiation of the B coefficient (Exp(B)) or odds ratio; ^4^ confidence interval; ^5^ reference category (RC) is female sex; ^6^ RC is ≤31 years of age; ^7^ RC is 0–7 years of working experience; ^8^ RC is no leading position; ^9^ RC is not working in acute care (predominantly in ICU and/or emergency department); ^10^ RC is the first/lowest tertile of the symptom score; ^11^ RC are lower sum scores based on median splits. *, extreme values (option “diverse” *n* = 2).
